# Oncogenic Properties of Apoptotic Tumor Cells in Aggressive B Cell Lymphoma

**DOI:** 10.1016/j.cub.2014.12.059

**Published:** 2015-03-02

**Authors:** Catriona A. Ford, Sofia Petrova, John D. Pound, Jorine J.L.P. Voss, Lynsey Melville, Margaret Paterson, Sarah L. Farnworth, Awen M. Gallimore, Simone Cuff, Helen Wheadon, Edwina Dobbin, Carol Anne Ogden, Ingrid E. Dumitriu, Donald R. Dunbar, Paul G. Murray, Dominik Ruckerl, Judith E. Allen, David A. Hume, Nico van Rooijen, John R. Goodlad, Tom C. Freeman, Christopher D. Gregory

**Affiliations:** 1 Medical Research Council (MRC) Centre for Inflammation Research, Queen’s Medical Research Institute, University of Edinburgh, Edinburgh EH16 4TJ, UK; 2 Institute of Infection and Immunity, School of Medicine, Cardiff University, Cardiff CF14 4XN, UK; 3 Paul O’Gorman Leukaemia Research Centre, Institute of Cancer Sciences, University of Glasgow, Gartnavel General Hospital, Glasgow G12 0XB, UK; 4 University of Edinburgh Departments of Haematology and Pathology, Western General Hospital, Edinburgh EH4 2XU, UK; 5 Centre for Cardiovascular Science, Queen’s Medical Research Institute, University of Edinburgh, Edinburgh EH16 4TJ, UK; 6 Cancer Research United Kingdom (CRUK) Institute for Cancer Studies, University of Birmingham, Birmingham B15 2TT, UK; 7 Centre for Immunity, Infection and Evolution, University of Edinburgh, Edinburgh EH9 3FL, UK; 8 The Roslin Institute, R(D)SVS, University of Edinburgh, Easter Bush EH25 9RG, UK; 9 Department of Molecular and Cell Biology, Free University Medical Centre, P.O. Box 7057, 1007 MB Amsterdam, the Netherlands

## Abstract

**Background:**

Cells undergoing apoptosis are known to modulate their tissue microenvironments. By acting on phagocytes, notably macrophages, apoptotic cells inhibit immunological and inflammatory responses and promote trophic signaling pathways. Paradoxically, because of their potential to cause death of tumor cells and thereby militate against malignant disease progression, both apoptosis and tumor-associated macrophages (TAMs) are often associated with poor prognosis in cancer. We hypothesized that, in progression of malignant disease, constitutive loss of a fraction of the tumor cell population through apoptosis could yield tumor-promoting effects.

**Results:**

Here, we demonstrate that apoptotic tumor cells promote coordinated tumor growth, angiogenesis, and accumulation of TAMs in aggressive B cell lymphomas. Through unbiased “in situ transcriptomics” analysis—gene expression profiling of laser-captured TAMs to establish their activation signature in situ—we show that these cells are activated to signal via multiple tumor-promoting reparatory, trophic, angiogenic, tissue remodeling, and anti-inflammatory pathways. Our results also suggest that apoptotic lymphoma cells help drive this signature. Furthermore, we demonstrate that, upon induction of apoptosis, lymphoma cells not only activate expression of the tumor-promoting matrix metalloproteinases MMP2 and MMP12 in macrophages but also express and process these MMPs directly. Finally, using a model of malignant melanoma, we show that the oncogenic potential of apoptotic tumor cells extends beyond lymphoma.

**Conclusions:**

In addition to its profound tumor-suppressive role, apoptosis can potentiate cancer progression. These results have important implications for understanding the fundamental biology of cell death, its roles in malignant disease, and the broader consequences of apoptosis-inducing anti-cancer therapy.

## Highlights

• Apoptotic lymphoma cells promote tumor growth, angiogenesis, and TAM accumulation
• Unbiased “in situ transcriptomics” analysis shows TAMs promote pro-tumor pathways
• Apoptotic tumor cells express and process matrix remodeling proteins
• The oncogenic potential of apoptotic tumor cells extends beyond lymphoma

## Graphical Abstract

**Figure:**
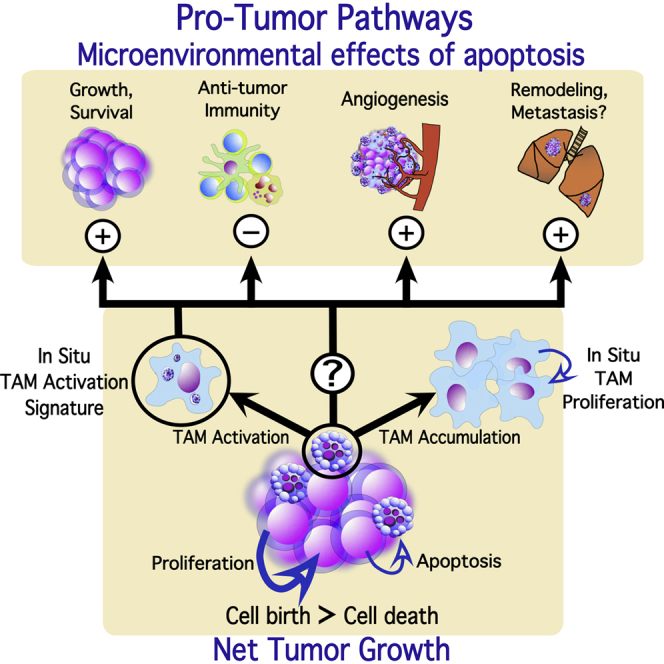

## Teaser

Apoptosis and tumor-associated macrophages (TAMs) are often associated with poor prognosis in cancer. Ford et al. demonstrate apoptotic lymphoma cells can promote tumor growth, angiogenesis, TAM accumulation, and TAM activation to potentiate cancer progression. These results have important implications for apoptosis-inducing anti-cancer therapies.

## Introduction

Cells dying by apoptosis are rapidly engulfed by phagocytes. Histologically, apoptotic cells are most commonly co-localized with macrophages, and the phagocytic response is accompanied by production of anti-inflammatory and trophic factors [1–4]. Similar tissue-reparatory activation states are typical of tumor-associated macrophages (TAMs), and there is growing recognition that TAMs often promote tumor growth and progression by facilitating angiogenesis, matrix remodeling, and metastasis and by suppressing anti-tumor immunity. Thus, TAM accumulation and activation are generally associated with poor prognosis. The pro-tumor properties of TAMs have been studied extensively in certain malignancies [5–7], but the mechanisms underlying oncogenic activation of TAMs are not fully understood.

Apoptosis has a defined purpose in preventing tumorigenesis [8], but, paradoxically, high incidence of apoptosis is linked to aggressive disease in multiple malignancies [9–14]. Indeed, cell loss is significant in aggressive tumors [9], and it is notable that programmed cell death can generate reparative and regenerative tissue responses such as angiogenesis and compensatory proliferation that have strong potential to be causally associated with tumor progression [4, 15].

Given the poor prognostic indications of both apoptosis and TAM content in malignant disease and the established functional relationship between apoptosis and macrophage activation, we hypothesized that loss of tumor cells by apoptosis and associated macrophage activation could facilitate progression of malignant disease. Here, we show that apoptosis promotes tumor growth, angiogenesis, and accumulation of pro-oncogenic TAMs in aggressive non-Hodgkin’s lymphoma (NHL).

## Results

### Suppression of Apoptosis in Lymphoma Cells Constrains Tumor Cell Proliferation In Vivo

**Figure 1:**
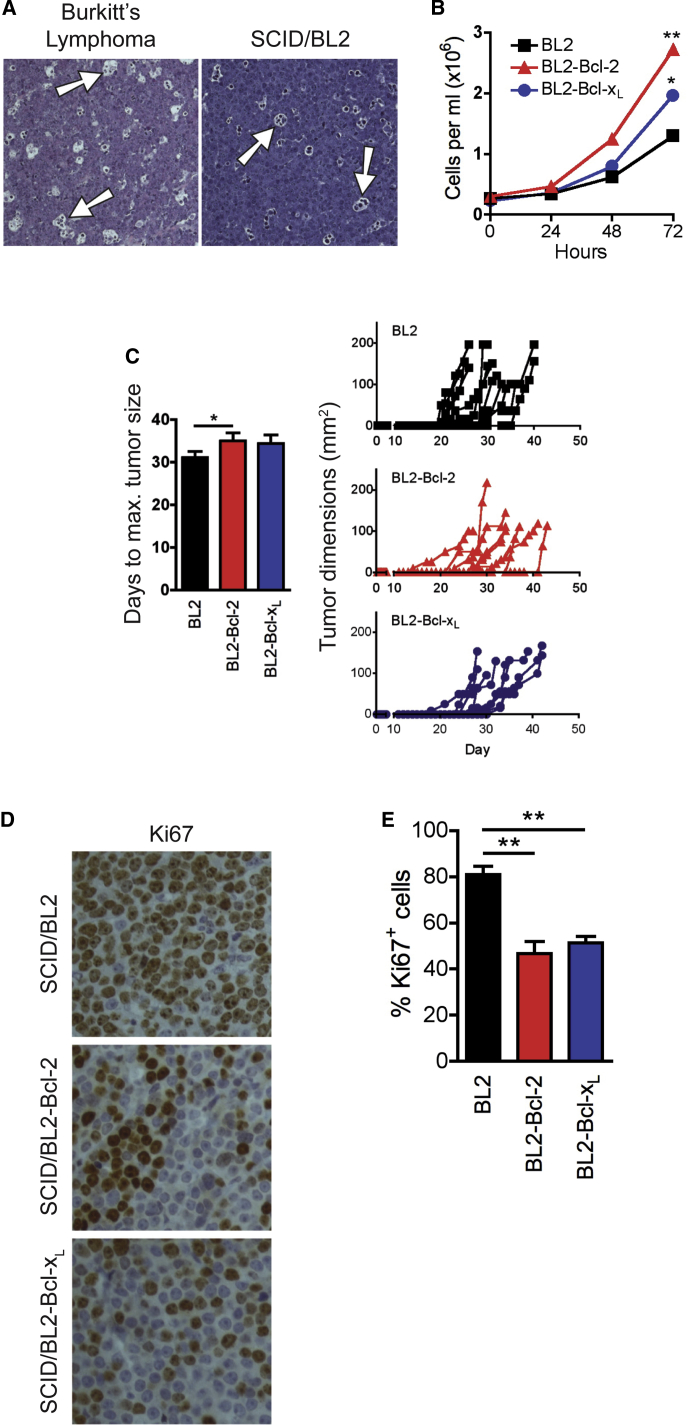
Suppression of Apoptosis in Lymphoma Cells Constrains In Vivo Proliferation and Angiogenesis (A) Representative H&E staining of tumors from BL patient (left) and SCID/BL2 xenograft (right) (n = 6). Arrows exemplify SS-TAMs. (B) Expression of exogenous *Bcl-2* or *Bcl-x*_*L*_ promotes expansion of BL2 cells in vitro. Means ± SEM (n = 3). ^∗∗^p = 0.0025, ^∗^p = 0.0328. Under normal growth conditions, typically, BL2 cultures are 90%, BL2-Bcl-2 cultures are 95%, and BL2-Bcl-x_L_ cultures are 93% AxV^−^PI^−^. (C) *Bcl-2* or *Bcl-x*_*L*_ gene expression fails to enhance BL2 xenograft growth rate. Mean days for tumor growth to 1.44 cm^2^ + SEM (left) and growth plots of all tumors (right) are shown; BL2 (n = 12), BL2-Bcl-2 (n = 8), BL2-Bcl-x_L_ (n = 8). ^∗^p = 0.0444. (D and E) Proliferation of BL is decreased in BL2-Bcl-2 and BL2-Bcl-x_L_ xenografts. Example of Ki67 IHC in each type of xenograft tumor is shown in (D). Quantification of Ki67 IHC in xenografts showing percent of Ki67^+^ cells per field + SEM (BL2 and BL2-Bcl-2: n = 8; BL2-Bcl-x_L_: n = 3) is shown in (E). ^∗∗^p = 0.0014 BL2-Bcl-2; ^∗∗^p = 0.0095 BL2-Bcl-x_L_.

We initially studied a xenograft model of an aggressive “starry-sky” NHL, Burkitt’s lymphoma (BL), in which apoptotic tumor cells are common and frequently observed in association with the starry-sky TAMs (SS-TAMs, so called because they appear histologically as “stars” in a “sky” of tumor cells) that accumulate in these tumors [16]. We used BL cell lines that phenotypically resemble the tumor biopsy cells from which they were derived, including the capacity to undergo apoptosis constitutively [17]. BL xenografts in severe combined immunodeficiency (SCID) mice closely recapitulate the starry-sky histological picture of the human lymphoma (Figure 1A). Apoptosis of lymphoma cells and their engulfment by SS-TAMs in situ was confirmed by immunohistochemistry (IHC; Figure S1). We first assessed whether apoptosis in lymphoma cells affects tumor growth. Suppression of apoptosis in BL cells through expression of anti-apoptotic Bcl-2 or Bcl-x_L_ promoted survival and expansion of transduced cell populations in vitro (Figure 1B). We previously demonstrated that expression of these proteins suppresses spontaneous and inducible apoptosis of lymphoma cells [18]. Remarkably, growth in vivo was not similarly improved by apoptosis suppression. In xenografts, apoptosis-suppressed BL cells showed no preferential capacity to form tumors, instead displaying an equivalent or slightly slower growth trend as compared to their “pro-apoptotic” parental counterparts (Figure 1C). Apoptosis-suppressed BL populations were markedly constrained in their capacity to proliferate in situ, displaying approximately half the levels of Ki67-positive cells as the parental populations in which apoptosis occurred constitutively (Figures 1D and 1E). These results indicate that suppression of apoptosis promotes autonomous survival of lymphoma cells but compromises additional pro-tumor mechanisms, which are otherwise generated by apoptotic B lymphoma cells in vivo.

### Apoptosis of B Lymphoma Cells Promotes Tumor Angiogenesis

**Figure 2:**
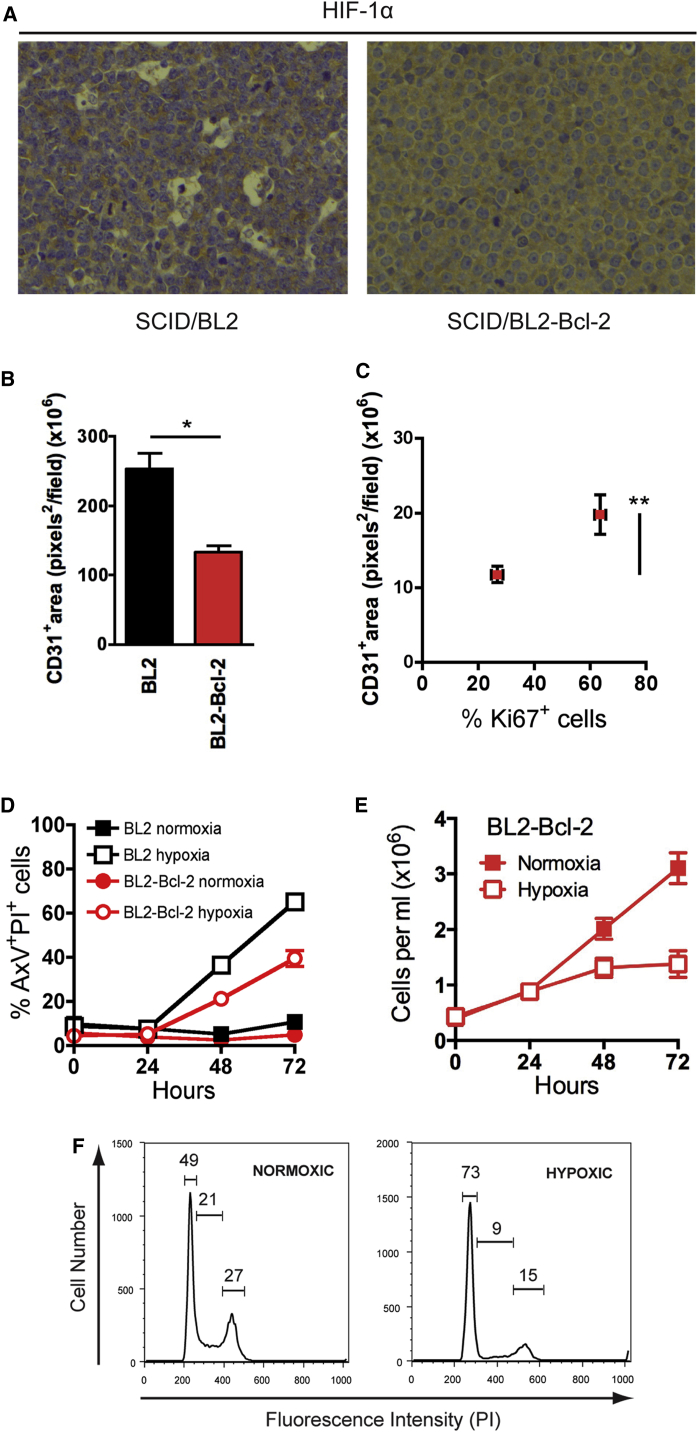
Apoptosis of Lymphoma Cells Promotes Tumor Angiogenesis (A) Representative HIF-1α IHC of xenograft tumors, BL2 (left) and BL2-Bcl-2 (right) (n = 5–6). (B and C) Reduced angiogenesis in tumors with suppressed apoptosis is associated with low cell proliferation. Quantification of CD31 IHC in BL2 and BL2-Bcl-2 xenograft tumors (mean pixels^2^ per field + SEM; n = 3–5) is shown (B). ^∗^p = 0.0184. Quantification of CD31 staining (pixels^2^ per field ± SEM) in BL2-Bcl-2 xenograft tumors in areas with high and low Ki67 expression (% per field ± SEM; n = 5; ^∗∗^p = 0.0061) is shown (C). (D and E) Hypoxia induces apoptosis in BL2 cells (D), whereas apoptosis-inhibited BL2-Bcl-2 cells survive but undergo growth arrest (E) in vitro. Mean ± SEM of three independent experiments. (F) Representative flow cytometric cell-cycle profiles of viable-gated BL2-Bcl-2 cells cultured for 48 hr (representative of three independent experiments).

Levels of HIF-1α were substantially lower in apoptosis-prone parental tumors, as compared to their Bcl-2-expressing counterparts (Figure 2A), suggesting that the former tumors were less hypoxic than the latter. Therefore, we investigated whether apoptosis promotes angiogenesis. We observed substantially reduced angiogenesis in apoptosis-suppressed xenografts as demonstrated by CD31^+^ endothelial cell density (Figure 2B). A strong association in BL2-Bcl-2 tumors between proliferation and CD31^+^ endothelial cell density was also evident (Figure 2C).

These results suggest that suppression of apoptosis in BL cells constrains the tumor cell population’s capacity to proliferate in vivo at least in part through reduced angiogenesis. We propose that hypoxia acts as a trigger for apoptosis in parental lymphoma cells but causes growth arrest in apoptosis-suppressed cells. In support of this, parental BL cells were induced into apoptosis under hypoxic conditions in vitro, whereas their Bcl-2-overexpressing counterparts survived better but underwent cell-cycle arrest (Figures 2D–2F).

### Apoptosis Promotes Macrophage Accumulation in Starry-Sky NHL

**Figure 3:**
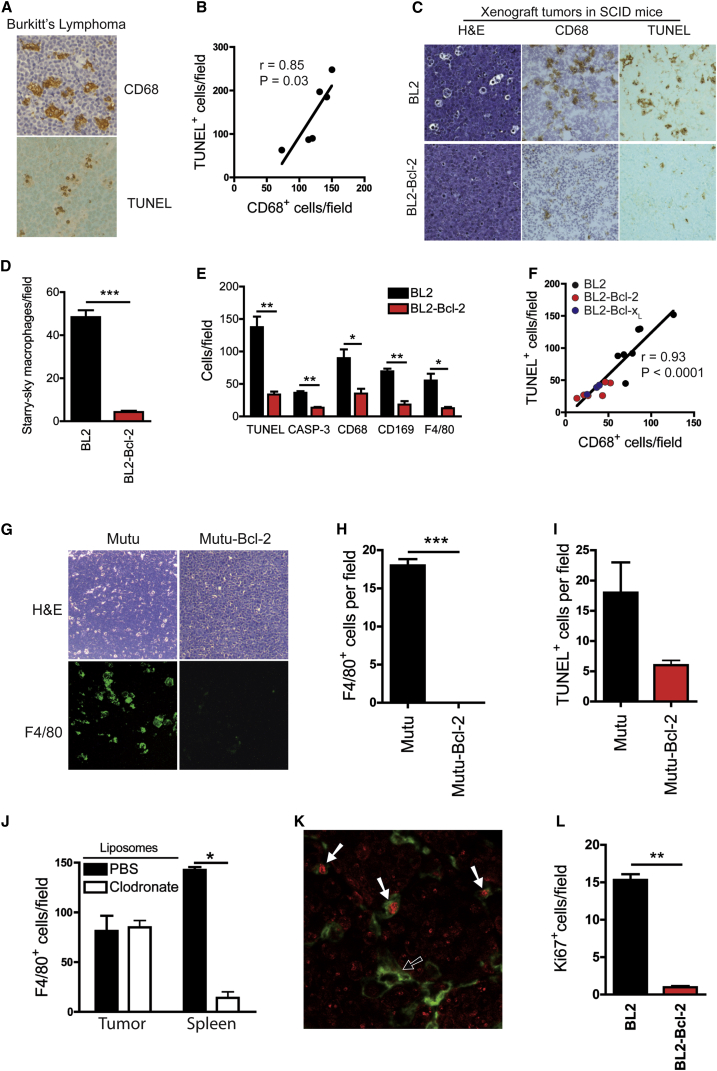
Apoptosis of Lymphoma Cells Promotes Macrophage Accumulation in Starry-Sky NHL, and This Is Associated with Macrophage Proliferation (A and B) Correlation between apoptosis and TAM accumulation in human BL. CD68 (TAM) and TUNEL (apoptosis) staining of BL biopsy is shown (A). Linear correlation of frequencies of CD68^+^ cells and TUNEL^+^ cells in BL (means per field; biopsies from six patients) is shown (B). (C–E) Parallel loss of starry-sky morphology, macrophage accumulation, and apoptosis incidence in BL2 xenografts expressing Bcl-2. Quantitative histochemical comparisons of starry-sky macrophages (H&E) (D), apoptosis (TUNEL and active caspase-3 [CASP-3]), and macrophage markers (CD68, CD169, and F4/80) (E) (n = 3–5; ^∗∗∗^p < 0.0001 H&E; ^∗∗^p = 0.0061 TUNEL; ^∗∗^p = 0.0061 active caspase-3; ^∗^p = 0.0152 CD68; ^∗∗^p = 0.0100 CD169; ^∗^p = 0.0259 F4/80; means + SEM) are shown. (F) Linear correlation of frequencies of TAMs (CD68^+^ cells) and apoptotic (TUNEL^+^) cells in BL2, BL2-Bcl-2, and BL2-Bcl-x_L_ xenografts (means per field; n = 17). ^∗∗∗^p < 0.0001. (G–I) Coordinated reduction of apoptosis and TAM accumulation in Mutu I BL xenograft tumors. Representative H&E for starry-sky appearance (G, upper panels) and for TAM accumulation (F4/80-FITC IHC) (G, lower panels) is shown. Morphometric analyses of TAM accumulation (H) and apoptosis (TUNEL staining, I) (n = 3). Means + SEM are shown. (J) Morphometric analysis of F4/80 IHC to determine frequency of F4/80^+^ cells in BL2 xenograft tumors and spleen from clodronate liposome-treated and control liposome-treated mice (n = 3). Means + SEM; ^∗^p = 0.0404. (K) Dual IHC of BL2 xenograft tumors for host Ki67^+^ (red) and F4/80^+^ (green) showing proliferating (white arrow) and non-proliferating (black arrow) TAMs (n = 3). (L) Mouse Ki67 IHC showing reduced incidence of Ki67^+^ host cells in apoptosis-suppressed BL2-Bcl-2 xenografts (n = 5). Means + SEM; ^∗∗^p = 0.0061. Dual staining for Ki67 and F4/80 showed 93.98% ± 1.3% (mean ± SEM) of proliferating host cells in BL2 xenograft tumors were F4/80^+^.

We hypothesized that the tumor-promoting and pro-angiogenic effects of apoptosis are due in part to activation of macrophages by apoptotic lymphoma cells. We determined whether apoptosis plays a role in controlling TAM accumulation in lymphomas. Observations of biopsies from BL patients showed that frequencies of apoptotic cells and TAMs were closely correlated (Figures 3A and 3B). In xenografts, suppression of apoptosis by Bcl-2/Bcl-x_L_ resulted in substantial reduction (though not abolition) of apoptosis. Parallel reduction in TAM density was also observed in apoptosis-inhibited tumors (Figures 3C–3I). This was unlikely to have been due to the apoptosis suppressors acting on alternative, apoptosis-independent mechanisms such as macrophage chemoattractant production (Figure S2).

To understand the mechanisms underlying TAM accumulation, we next tested the effect of liposomal clodronate, which is known to reduce tissue macrophage numbers through blockade of blood-monocyte recruitment [19]. Intravenous liposomal clodronate failed to deplete TAMs despite depleting splenic macrophages effectively (Figure 3J). Moreover, we found significant numbers of proliferating TAMs in parental xenografts (Figure 3K). Notably, xenografts in which apoptosis was suppressed by Bcl-2 displayed substantially less proliferation of all host cells (Figure 3L).

These results show that SS-TAM accumulation, in part occurring via macrophage proliferation in situ, is promoted by constitutive lymphoma cell apoptosis, which is coupled to enhanced tumor growth and angiogenesis.

### Multiple Pro-tumor Pathways Are Activated in SS-TAMs

Since tumor cell apoptosis caused the accumulation of SS-TAMs, we reasoned that apoptotic lymphoma cells further engage with SS-TAMs to activate diverse tumor-growth-promoting pathways. Since macrophages are highly responsive to their environments, we decided against isolating SS-TAMs, which risks changing their transcriptional profile during the necessary tissue destruction processing. Rather, we investigated global gene expression signatures of undisturbed SS-TAMs in their natural habitat. We adopted laser-capture microdissection of individual SS-TAMs in BL xenografts in order to obtain unbiased in situ transcriptional profiles of these cells, which we compared specifically with those of similarly captured macrophages, the tingible-body macrophages from normal germinal centers (GCMs) (Figure S3A). The rationale for this comparison was based upon BL being a germinal center malignancy and tingible-body macrophages being regarded as normal equivalents of SS-TAMs.

**Figure 4:**
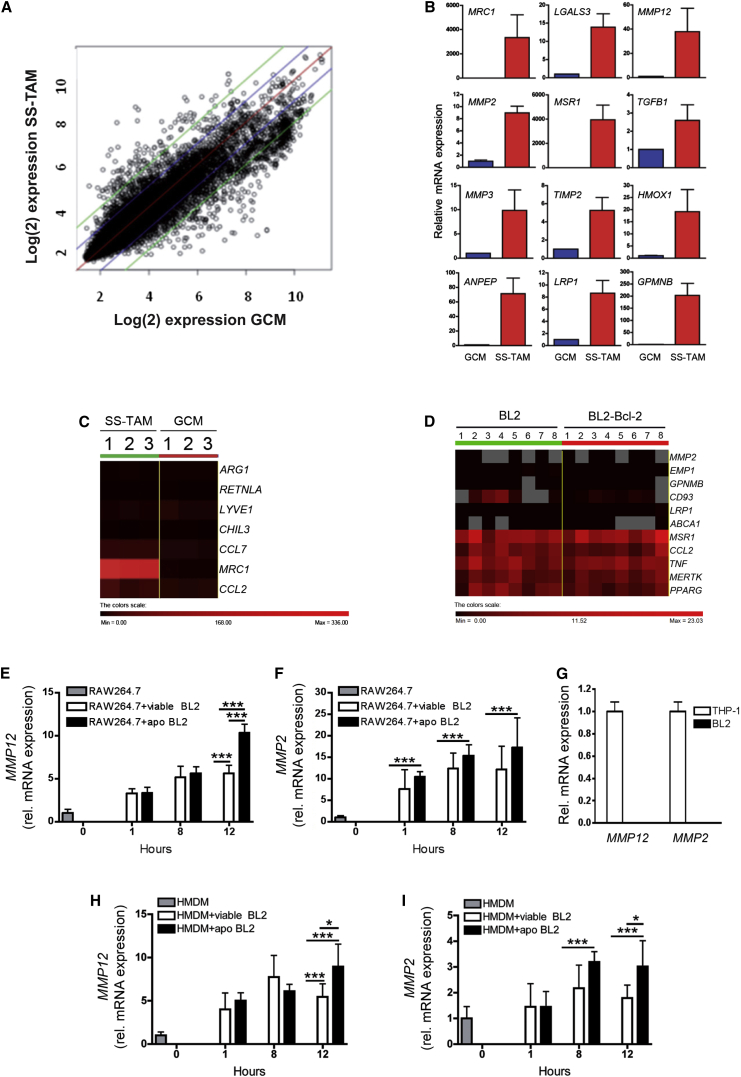
Pro-tumor Activation Status of SS-TAMs (A) Scatterplot comparison of mean log fluorescence intensity of each SS-TAM versus each GCM transcript. Gene expression changes within: red lines indicate no change, blue indicates ±2-fold changes, and green indicates ±4-fold changes (n = 3 animals). (B) Validation of selected genes from SS-TAMs and GCMs by qPCR. Means + 95% confidence intervals for n = 2. (C) Heat map comparing in situ transcriptome of prototypic alternative activation gene signatures of SS-TAMs and GCMs (n = 3 animals). (D) Heat map showing Fluidigm gene expression analysis of genes in BL2 compared to BL2-Bcl-2 xenograft formalin-fixed, paraffin-embedded tumor tissue (n = 8). Data were normalized to *CD68* gene expression. (E and F) qPCR showing upregulation of *MMP12* (E) and *MMP2* (F) mRNA in RAW264.7 macrophages following co-culture with viable or apoptotic (apo) BL2 lymphoma cells (BL2 apoptosis was induced by serum starvation for 1 hr) (n = 3). ^∗∗∗^p < 0.001 (E); ^∗∗∗^p < 0.001 (F). Means + SEM. (G) qPCR showing absence of *MMP12* and *MMP2* mRNA expression in BL2 cells. Data were normalized to expression by THP-1 cells (n = 3). Means + SEM. (H and I) qPCR showing upregulation of *MMP12* (H) and *MMP2* (I) mRNA in HMDM macrophages following co-culture with viable or apoptotic (apo) BL2 lymphoma cells (BL2 apoptosis was induced by serum starvation for 1 hr, 30%–39% AxV^+^/PI^-^) (n = 3). Relative mRNA expression shown is mean fold change (+SEM) compared to HMDM control. ^∗∗∗^p < 0.001, ^∗^p < 0.05 (H); ^∗∗∗^p < 0.001, ^∗^p < 0.05 (I).

Scatterplot analyses of microarray data (Figure 4A) indicated that, when SS-TAMs were compared with GCMs, 398 genes were upregulated and 997 were downregulated. In accordance with previously proposed paradigms for macrophage activation in tumors, the transcriptome of SS-TAMs in parental BL xenografts was found to reflect an anti-inflammatory, reparatory pro-angiogenesis and tissue-remodeling activation state (selected genes from microarrays are shown in Table 1; validation by qPCR is shown in Figures 4B and S3). Fidelity of macrophage transcripts obtained via laser-capture microdissection was confirmed (Figures S3B and S3C). Several of the genes associated with the IL-4Rα-dependent “alternative activation” state, including *ARG1*, were either not expressed or only expressed at low levels by SS-TAMs (Figure 4C). Others were markedly upregulated, notably *CD206* (*MRC1*), which showed the highest relative expression (38-fold increase compared to GCMs) in SS-TAMs (Table 1, asterisks).

Network analysis [23, 24] indicated that SS-TAMs exhibited features of various macrophage types and activation states, including peritoneal and bone marrow-derived macrophages (Figure S3D). Examination of the most highly expressed transcripts identified enrichment of numerous functional pathways in SS-TAMs that were mainly associated with enhanced phagocytic capacity, elevated lipid metabolism, matrix remodeling, and wound healing (Figures S3E and S3F). The phagocytic pathway results confirm the active clearance of apoptotic cells by these macrophages that is evident in the histological picture. Genes encoding receptors, bridging molecules and anti-inflammatory mediators known to be involved in recognition of and responses to apoptotic cells, were among the transcripts most highly enriched in SS-TAMs. These include *MSR1*, *CD36*, *LRP1*, *CD93*, *MERTK*, *GAS6*, *TREM2*, *PPARγ*, and the most renowned anti-inflammatory mediator produced by phagocytes in response to apoptotic cells, *TGF-β1* (Table 1).

Strikingly, strong expression of genes encoding growth factors IGF-1 and PDGF-CC (Table 1), which have been shown potently to protect cells from apoptosis [25, 26], firmly indicates that SS-TAMs provide trophic cues in the lymphoma microenvironment with the potential to promote net population growth. In accordance with their multi-functional tumor-promoting capacity, SS-TAMs were further found to highly express genes with capacity to promote angiogenesis and matrix remodeling (Table 1; Figures 4B and S3C), including transcripts encoding MMP2, 3, and 12, TIMP-2, CD13, galectin-3, heme oxygenase-1, and GPNMB, which are strongly implicated in these processes [27–30].

### Apoptotic Lymphoma Cells Drive Macrophage MMP2 and MMP12 Expression

We next studied the relationship between lymphoma cell apoptosis and TAM activation. With reference to selected genes from the unbiased profiling of SS-TAMs from parental BL xenografts, we found that the activation profiles of TAMs from parental as compared to Bcl-2 tumors were similar (Figures 4D and S4). Therefore, since apoptosis was reduced rather than abolished, in Bcl-2-overexpressing BL tumors (Figures 3E, 3F, and 3I), the (albeit smaller) contingent of TAMs in these tumors could be activated similarly to parental SS-TAMs via interaction with the smaller numbers of apoptotic cells present. Alternatively, the activation status of accumulated TAMs could be influenced by apoptosis-independent properties of the lymphoma cells. In order to differentiate between these possibilities, we compared co-culture of macrophages in vitro with viable versus apoptotic lymphoma cells. We chose to investigate macrophage gene expression associated with matrix degradation—a significant facet of tumor growth and progression—since no relationship between the latter and macrophage interaction with apoptotic cells has been previously established. We observed marked upregulation of both *MMP2* and *MMP12* by apoptotic lymphoma cells (Figures 4E–4I). Viable lymphoma cells may also contribute to *MMP2* and *MMP12* upregulation, but it is noteworthy that “viable” populations contained small numbers of constitutively apoptotic cells.

### Apoptosis Promotes Tumor Growth in an Alternative Model of Aggressive NHL

**Figure 5:**
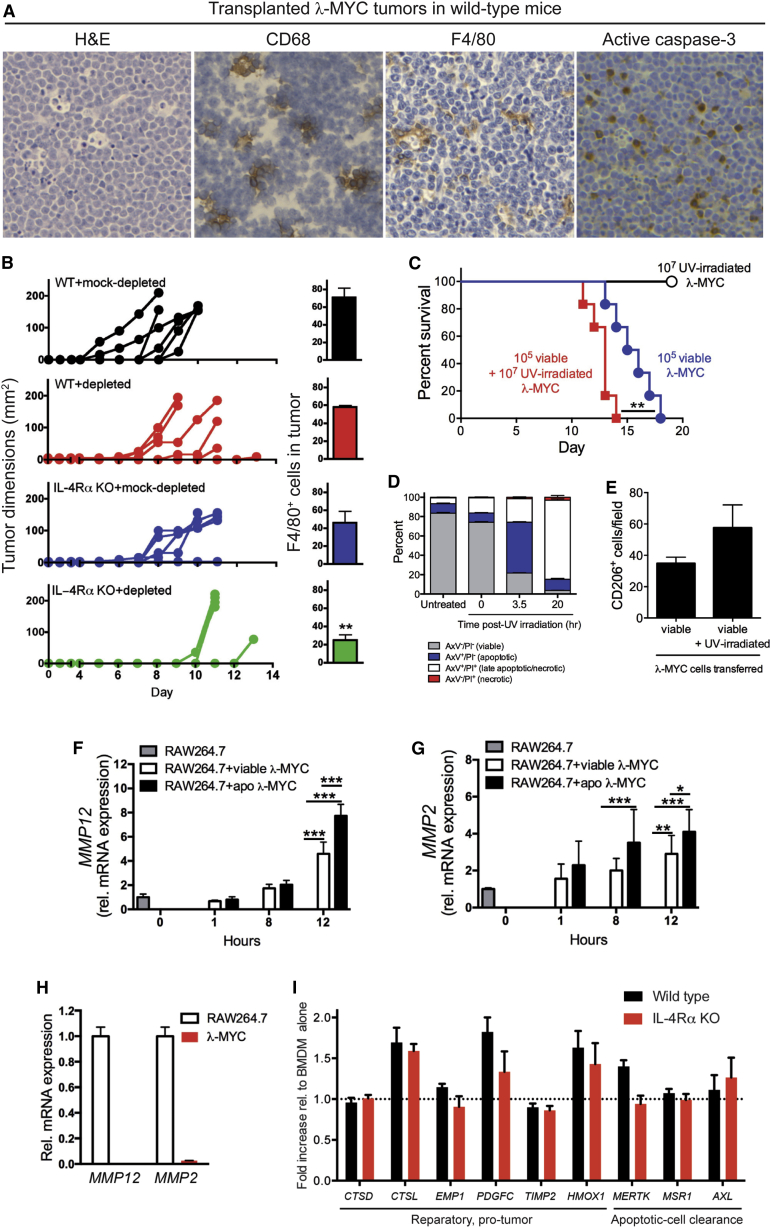
The Growth of Syngeneic λ-MYC Transplanted Tumors Can Be Modified by Regulation of Tumor Cell Apoptosis (A) Typical starry-sky histology in syngeneic λ-MYC transplanted tumors in C57BL/6 mice. Representative H&E-stained, CD68-stained, F4/80-stained, and active caspase-3-stained sections are shown. (B) Syngeneic λ-MYC lymphoma growth and incidence of F4/80^+^ TAM in WT and IL-4Rα KO. C57BL/6 mice received 5 × 10^6^ viable λ-MYC cells at day 0 in the presence of 3.1 × 10^6^ (mock-depleted sample) or 0.3 × 10^6^ (depleted sample) apoptotic λ-MYC cells (n = 5–6 animals for tumor growth and n = 3–5 animals for F4/80; means + SEM). ^∗∗^p = 0.0061. (C) Kaplan-Meier plots for C57BL/6 mice following transfer of 1 × 10^5^ viable λ-MYC cells alone, co-injected with 1 × 10^7^ UV-irradiated (200 mJ/cm^2^) λ-MYC cells or UV-irradiated cells alone (six animals per group). ^∗∗^p = 0.0076. (D) UV-irradiated λ-MYC cells underwent apoptosis in vitro as assessed by AxV/PI staining. Means + SEM for three independent experiments. (E) Morphometric analysis of resultant λ-MYC tumors immunostained for CD206-expressing cells (means + SEM; n = 5). No significant differences were observed. (F–H) qPCR showing upregulation of *MMP12* (F) (^∗∗∗^p < 0.001) and *MMP2* (G) (^∗∗∗^p < 0.001, ^∗∗^p < 0.01, ^∗^p < 0.05) mRNA in RAW264.7 macrophages following co-culture with viable or apoptotic (apo) λ-MYC (λ-MYC) lymphoma cells (λ-MYC apoptosis was induced by serum starvation for 1 hr, AxV^+^/PI^-^ cells 25%–30%) (n = 3). *MMP12* and *MMP2* mRNA was restricted to the macrophages in the co-cultures since little or no expression was detected in λ-MYC cells by real-time qPCR (H). Data were normalized to *TUBA1B* endogenous levels. All values are means + SEM of three independent experiments. (I) WT versus IL-4Rα KO BMDM responses to apoptotic λ-MYC cell co-culture. BMDM from WT or IL-4Rα KO C57BL/6 mice were co-cultured for 24 hr ± apoptotic λ-MYC cells. Apoptosis of λ-MYC cells was triggered by 100 mJ UVB irradiation. mRNA expression of selected genes was assessed following co-culture and normalized using *HSP90*, *TUBA1B*, and *HPRT*. Expression is presented as mean fold change + SEM compared to BMDM control.

We wished to determine whether the principle of tumor cell apoptosis as a promoter of tumor growth could be extended to other models. We first investigated a model of aggressive B cell lymphoma in immunocompetent mice, λ-MYC lymphoma [31], which also displays starry-sky features with frequent TAMs and apoptotic events (Figure 5A). The use of a starry-sky lymphoma model in immunocompetent, wild-type (WT) mice additionally provided the opportunity to study the role of IL-4Rα, since several of the genes upregulated in SS-TAMs were IL-4Rα dependent (Table 1, asterisks). We derived and selected λ-MYC B lymphoma lines that, like the BL lines, retained the phenotypic characteristics, including pro-apoptotic propensity, of the tumor biopsy cells. When grafted to WT mice, these lines formed rapidly growing tumors (Figure 5B, upper panel). Upon removal of apoptotic cells from the transplant inoculum, we observed delayed or inhibited tumor growth (Figure 5B). Conversely, UV irradiated (apoptosis-committed) λ-MYC cells admixed with viable lymphoma cells in the transplant inoculum promoted tumor growth (Figures 5C and 5D). Despite no significant differences in accumulating TAMs in WT animals, IL-4Rα^−/−^ mice displayed delayed tumor growth (Figures 5B and 5E). Most strikingly, the combination of removal of apoptotic lymphoma cells from the graft inoculum together with IL-4Rα deficiency led to the most marked inhibitory effect on tumor growth and also to a significant reduction in TAM accumulation (Figure 5B, lower panel).

Like BL cells, λ-MYC lymphoma cells, especially when apoptotic, were able to activate *MMP2* and *MMP12* expression in macrophages in vitro (Figures 5F–5H) and promote the expression of a variety of SS-TAM markers in bone marrow-derived macrophages (BMDMs) (Figures S5A and 5I). Furthermore, exposure of classically activated BMDMs to apoptotic cells inhibited production of pro-inflammatory cytokines (Figures S5B and S5C). Notably, none of the modulatory effects induced by apoptotic lymphoma cells required macrophage IL-4Rα, indicating that the tumor-promoting effects of apoptotic λ-MYC cells and global IL-4Rα may be independent. This possibility is supported by the additive inhibitory effects on tumor growth and macrophage accumulation observed both when apoptotic cells are removed from the graft and when IL-4Rα is deficient in the host (Figure 5B).

### Apoptosis Triggers Direct Activation of MMP2 and MMP12 in B Lymphoma Cells

Given the pleiotropic functions of MMPs in tumor growth and/or progression and the consistent upregulation of *MMP2*/*12* in macrophages exposed to apoptotic lymphoma cells, we began analyzing the expression and processing of macrophage MMP2 and MMP12 at the protein level. Remarkably, in the course of these studies, we observed that apoptotic lymphoma cells directly upregulated and processed MMP2 and MMP12 polypeptides in the absence of other cell types in both murine and human models (Figures S5D–S5F). These results illustrate that the apoptosis program of lymphoma cells has the potential to drive tumor progression via MMP2/12 activation not only through TAM accumulation and activation but also via alternative mechanisms based on production and processing of these MMPs directly by the apoptotic lymphoma cells.

### Apoptosis Promotes Tumor Growth in a Non-lymphoma Model

**Figure 6:**
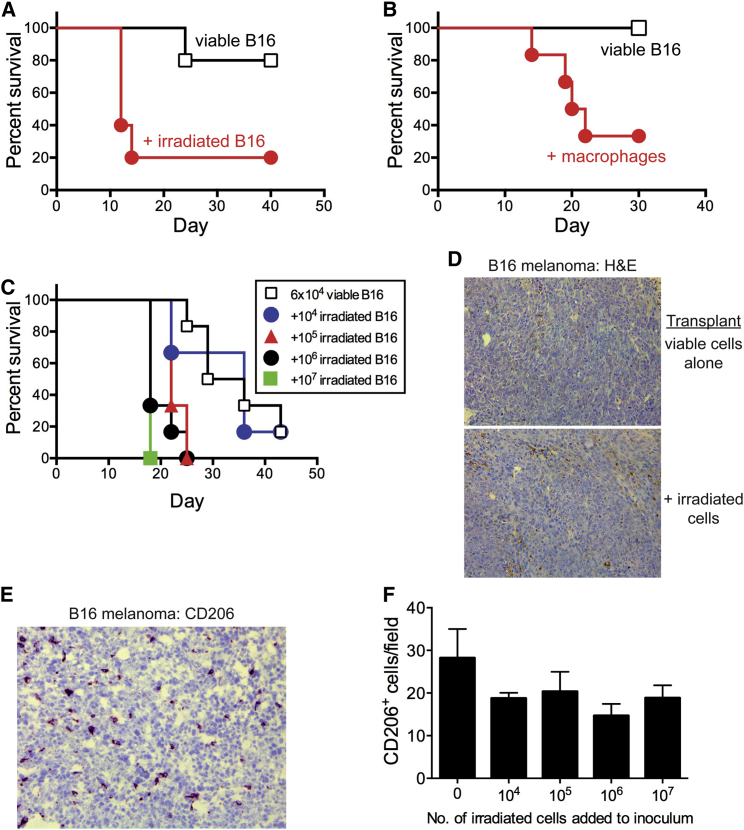
Apoptosis Promotes Tumor Growth in a Non-lymphoma Model (A–C) Kaplan-Meier plots for C57BL/6 mice following transfer of 6 × 10^4^ viable B16 cells alone and co-injected with irradiated B16 cells (10 × 10^6^) (A), syngeneic peritoneal macrophages (B), and irradiated B16 cells (various doses up to 10 × 10^6^) (C). Irradiated B16 cells underwent apoptosis in vitro as described previously [32] and failed to grow in vivo (data not shown). p = 0.031, n = 5 (A); p = 0.018, n = 6 (B); irradiated B16 cells, 0.1 × 10^6^: p = 0.0022, 1 × 10^6^: p = 0.0011, 10 × 10^6^: p = 0.0009, n = 6 (C). (D) H&E staining of B16 melanoma tumors generated by injection of 6 × 10^4^ viable cells ± 10 × 10^6^ irradiated B16 cells. (E) Representative image of CD206 IHC in B16 melanoma. (F) Morphometric analysis of CD206 IHC in B16 melanoma generated by injection of 6 × 10^4^ viable cells with various numbers of irradiated cells (means + SEM; n = 3–5).

Since apoptosis is linked to poor prognosis in a range of cancer types, we determined in principle whether apoptosis-driven tumor growth is restricted to NHL or whether it has a more general cancer-promoting role. We therefore analyzed the effect of tumor cell apoptosis on tumor growth in B16 melanoma. The presence of apoptotic tumor cells in the transplantation inoculum greatly enhanced the aggressiveness of this tumor in a dose-dependent manner (Figures 6A and 6C) without changing the architecture of the tumor (Figure 6D). Similarly enhanced aggressiveness was observed when viable peritoneal macrophages, which share certain transcriptional features with SS-TAMs (Figure S3D), were mixed with the tumor inoculum (Figure 6B). B16 melanomas harbored numerous CD206^+^ TAMs (Figure 6E), although significant differences in TAM numbers between tumors produced in the presence or absence of admixed tumor cells were not observed (Figures 6D–6F). These results indicate that apoptosis of tumor cells constitutes a mechanism to promote progression of cancer that extends beyond NHL.

## Discussion

It has been rigorously established that apoptosis is important in inhibiting malignant disease [8]. Less intuitively, apoptosis of tumor cell populations has the potential to contribute to oncogenic processes through multiple mechanisms based on several known properties of apoptotic cells, notably those that affect the tissue microenvironment and the immune system [4]. Here, we demonstrate that the constitutive apoptosis occurring in high-grade B cell lymphoma displays pro-tumor activities by promoting angiogenesis and the accumulation of tissue-reparatory and growth-promoting macrophages. We report the first genome-wide transcriptomics analyses of tissue macrophages interacting with apoptotic cells in situ, specifically apoptotic B cells in normal and malignant environments. Our results demonstrate that apoptosis in high-grade malignant lymphoma fosters the accumulation of macrophages that activate diverse pro-tumor signaling pathways. We propose that sustained apoptosis of a minor component of the lymphoma cell population promotes tumor growth and progression. In this regard, the macrophages respond to apoptosis in the “homeostatic mode” that is featured in normal development and tissue repair; in the context of cancer, this normal host response is “hijacked” for the benefit of the rogue, malignant tissue. In addition to the effects of apoptotic lymphoma cells on TAMs, we also report that apoptosis-induced human and murine lymphoma cells upregulate and process MMP2 and MMP12. While assessment of the significance of this observation for the oncogenic process will require substantial further investigation, it is tempting to speculate that conserved pro-tumor mechanisms can be generated by activation of the apoptosis program of the lymphoma cell. Our findings provide a mechanistic basis for the strong correlation between high apoptosis index, lack of Bcl-2 expression, and poor prognosis in NHL, regardless of histological type [10].

This study has focused on B cell lymphoma, but, given the association of aggressive disease with high apoptosis indices and macrophage frequency in multiple cancer types [9–14], it seems likely that the mechanisms reported here will prove significant in other malignancies, too. Results presented here using a syngeneic B16 melanoma transplant model extend a recent report of similar effects in an allogeneic setting [33] and support the proposition that apoptosis drives pro-tumor activity outside the lymphoma arena. The importance and route of apoptosis-driven mechanisms may prove to be context dependent, with variation between tumor types (and perhaps individual tumors). For example, constitutive pro-inflammatory TAM activation is a feature of the most common human sarcoma, gastrointestinal stromal tumor (GIST), and these TAMs constrain tumor growth. However, imatinib-induced apoptosis of tumor cells in GIST therapy appears to cause tumor-promoting activation of the TAMs and ultimately leads to imatinib resistance [34].

Our findings extend the known reparatory, immunosuppressive, and anti-inflammatory impacts of macrophages interacting with apoptotic cells [1–4], as well as developmental consequences of apoptosis such as compensatory proliferation and apoptosis-induced wound healing and regenerative responses [15], providing a rationale for cell death promoting oncogenic progression or relapse post-treatment. While anti-cancer therapies that induce apoptosis in tumor cells drive the cell birth/cell death equation toward cure, it is becoming clear that apoptosis-inducing therapeutic effects can lead to tumor re-population [35]. These findings, taken together with those presented here, emphasize the need for therapeutic modalities, such as immunogenic tumor cell-death-inducing treatments [36] that simultaneously activate host anti-tumor responses as well as cell death pathways.

Although the molecular mechanisms critical for pro-tumor activation of SS-TAMs by apoptotic cells require further evaluation, the present work highlights several candidate oncogenic players such as galectin-3 and the TYRO/AXL/MER axis. Intriguingly, it was recently demonstrated in breast cancer that the widespread tumor cell death and accompanying macrophage-mediated clearance and activation, which is associated with post-partum mammary gland involution, promoted tumor metastasis and was dependent upon MER tyrosine kinase [37].

Angiogenic and growth/survival factors such as IGF-1 are induced in SS-TAMs, and our results are consistent with tumor cell apoptosis caused by hypoxia or growth/survival factor deprivation promoting angiogenesis or trophic factor production via TAM activation. Our investigations into the IL-4/IL-13 axis indicated that global expression of IL-4Rα was required for optimal pro-tumor effects of apoptotic lymphoma cells, as well as optimal TAM accumulation (Figure 5B). The apoptotic cells were capable of directly activating macrophages in vitro toward a pro-tumor phenotype (and away from an anti-tumor pro-inflammatory state) in the absence of IL-4Rα. This suggests that the role of the IL-4/IL-13 axis in the pro-tumor effects of apoptosis is distinct from the IL-4Rα-dependent, alternative macrophage activation pathway. A similar activation signature that was distinct from alternative macrophage activation (with the notable exception of *MRC1*) was recently reported for TAMs in a breast cancer model [6]. Taken together with our observation that apoptotic cells and IL-4Rα have additive effects in promoting tumor growth (and macrophage accumulation), our results are consistent with roles for IL-4/IL-13 in driving NHL-promoting mechanisms independently of apoptosis. Our findings (Figures 5 and S5) strongly indicate that apoptotic lymphoma cells do not activate macrophages via this route. This conclusion is further supported by our observations that neither human nor murine NHL cells studied here secreted IL-4 or IL-13 either constitutively or following induction of apoptosis.

Given the heterogeneity of tumor cell populations and stromal elements in cancer together with the plasticity of tissue macrophages, it is not surprising that signatures of TAMs in different tumor types have not proved entirely consistent, although the general paradigm of activation toward a reparatory, anti-inflammatory gene expression pattern holds for tumor-promoting TAMs in different models. It seems reasonable to assume that specific activation pathways are likely to be important for some tumors, but not others. Thus, it has recently been proposed for certain syngeneic transplantable solid murine tumors that lactate production by the tumor cells generates a conserved, “alternative activation-like” TAM signature that is orchestrated by HIF-1α and characterized by arginase-1 and VEGF activation [7]. In our xenograft model, we found neither arginase-1 (Figures 4C and S4B) nor VEGF (unpublished data) to be activated in SS-TAMs. The extent to which lactate plays a role in activating TAMs in NHL remains to be determined, as does the generality and molecular detail of the apoptosis-driven TAM activation mechanism(s).

The results presented here raise many questions concerning the impact of cell death—either that of the constitutive fraction of growing tumor cells or that induced by therapy—on the pathogenesis of cancer and on the role of macrophages in promoting tumor growth. Furthermore, the tumor-promoting mechanisms emanating from apoptosis of transformed cells may significantly influence other immune and non-cancerous host cells as well as transformed relatives (notably cancer stem cells). It is well established that mitogenic signals, such as Wnt, Hedgehog, and prostaglandin E_2_, are produced by apoptotic cells of diverse metazoans during development, in response to injury and during regeneration, including re-population of tumors after radiotherapy [15, 35, 38]. Given the observed mechanistic similarities between cancer, wound healing, and tissue regeneration, it seems likely that apoptosis may play related roles in all three. It is already clear that apoptosis-induced compensatory proliferative mechanisms are variable and context dependent. Therefore, the apoptosis-mediated mechanisms relevant to tumor evolution and growth seem likely to be similarly complex. Our results demonstrate that responses of phagocytes are a key part of this complexity (as previously proposed [4, 39]) and that cell death and tissue remodeling are closely interwoven in NHL. Further research will be required to investigate these new directions, and, based on the present findings, a full understanding of the pro-tumor effects of apoptosis in different types of cancer is warranted.

## Experimental Procedures

Experimental protocols including in situ transcriptomics methods developed for these studies are detailed in the Supplemental Information.

### Statistics

Statistical significance was determined using Wilcoxon matched pairs (Figure 3H), Mann-Whitney nonparametric tests (Figures 1B, 1C, 1E, 2B, 2C, 3D, 3E, 3J, 3L, and 5B), or one-way ANOVA followed by Tukey-Kramer post-test (Figures 4E, 4F, 4H, 4I, 5F, and 5G). Statistical significance of gene expression analyses was tested using LIMMA software or the Benjamini and Hochberg procedure for multiple testing corrections. Significance is represented as ^∗^p < 0.05, ^∗∗^p < 0.01, and ^∗∗∗^p < 0.001. Kaplan-Meier plots were compared using the Mantel-Cox log rank test (Figures 5C and 6A–6C). For animal studies, sample sizes were guided by previous experimentation [16, 40, 41].

## Author Contributions

C.A.F., S.P., J.D.P, J.J.L.P.V., L.M., M.P., S.L.F., A.M.G., S.C., H.W., E.D., C.A.O., and I.E.D. designed and performed the experiments. T.C.F. and D.R.D. advised and assisted with bioinformatics. P.G.M. and J.R.G. provided advice and archived tumor tissue. D.R., J.E.A., and D.A.H. provided advice and mice. N.v.R. provided clodronate/PBS liposomes. C.D.G. conceived the project, designed the experiments, and provided overall coordination. C.D.G., C.A.F., J.D.P., and S.L.F. wrote the manuscript, with input from other authors.

## Figures and Tables

**Table 1:** Selected Transcripts Preferentially Expressed in SS-TAMs as Compared with GCMs

Gene	Protein	Fold ↑	p Value
**Genes Associated with Reparatory and Pro-tumor Macrophage Responses**			

*MRC1^∗^*	mannose receptor, C type 1 (CD206)	38	<0.0001
*ANPEP^∗^*	alanyl (membrane) aminopeptidase (CD13)	16.6	<0.0001
*GPNMB*	glycoprotein (transmembrane) NMB (osteoactivin)	8.8	0.00060
*PLAU*	urokinase plasminogen activator	6.7	0.00011
*CTSD*	cathepsin D	6.4	0.00081
*CTSB*	cathepsin B	5.8	<0.0001
*FN1^∗^*	fibronectin 1	5.7	0.00903
*HMOX1*	heme oxygenase (decycling) 1	5.5	0.00195
*CTSL*	cathepsin L	5.4	0.00103
*PSAP*	prosaposin	4.2	0.00105
*TIMP2^∗^*	tissue inhibitor of metalloproteinase 2	4	0.00022
*MMP12^∗^*	matrix metalloproteinase 12	3.8	0.04470
*MMP3*	matrix metalloproteinase 3	3.5	0.04072
*IGF1^∗^*	insulin-like growth factor 1	2.5	0.00014
*SEPP1^∗^*	selenoprotein P plasma 1	2.7	0.00046
*EMP1*	epithelial membrane protein 1	2.3	0.00018
*PDGFC*	platelet derived growth factor CC	2.3	0.00843
*MMP2*	matrix metalloproteinase 2	2.1	0.00072
*CTSS*	cathepsin S	2.1	0.00063
*LAMP2*	lysosomal associated membrane protein 2	2	0.00016
*CCL2^∗^*	chemokine (C-C motif) ligand 2	2	0.01138

**Genes Associated with Macrophage Responses to Apoptotic Cells**			

*MSR1*	macrophage scavenger receptor (CD204)	17	<0.0001
*LRP1*	LDL receptor-related protein (CD91)	8.4	<0.0001
*MERTK*	c-Mer tyrosine kinase	7.5	<0.0001
*CD36*	CD36 scavenger receptor	5.9	0.00375
*CD93^∗^*	CD93 receptor	5.9	0.00162
*LGALS3*	galectin 3	5.2	<0.0001
*ABCA1^∗^*	ATP-binding cassette transporter	5.2	<0.0001
*PPARG*	peroxisome proliferator activated receptor gamma	4.1	0.00115
*AXL^∗^*	AXL receptor tyrosine kinase	3.9	0.00020
*TREM2*	triggering receptor expressed on myeloid cells 2	3.5	<0.0001
*GAS6^∗^*	growth arrest specific 6	2.7	0.00578
*TGFB1*	transforming growth factor beta 1	2.3	0.00476

Transcripts preferentially expressed in SS-TAMs as compared with GCMs and associated especially with reparatory and pro-tumor activation states, including trophic/angiogenesis/remodeling pathways, and engagement with apoptotic cells are shown here. Asterisks indicate genes also associated with IL-4Rα-dependent, alternative activation [20–22]. Data are from three animals.

## Accession Numbers

The GEO [42] accession number for the microarray data reported in this paper is GSE64366.

## Supplemental Information

Document S1. Supplemental Experimental Procedures and Figures S1–S5

Document S2. Article plus Supplemental Information
