# Supplementary material for: Oncogenic Properties of Apoptotic Tumor Cells in Aggressive B Cell Lymphoma
Source: Curr Biol. 2015 Mar 2;25(5):577–88. doi: 10.1016/j.cub.2014.12.059 (PMC4353688; doi:10.1016/j.cub.2014.12.059)
Supplement: Document S1. Supplemental Experimental Procedures and Figures S1–S5 [file mmc1.pdf]

Current Biology

Supplemental Information

Oncogenic Properties of Apoptotic Tumor Cells in Aggressive B Cell Lymphoma

Catriona A. Ford, Sofia Petrova, John D. Pound, Jorine J.L.P. Voss, Lynsey Melville, Margaret Paterson, Sarah L. Farnworth, Awen M. Gallimore, Simone Cuff, Helen Wheadon, Edwina Dobbin, Carol Anne Ogden, Ingrid E. Dumitriu, Donald R. Dunbar, Paul G. Murray, Dominik Ruckerl, Judith E. Allen, David A. Hume, Nico van Rooijen, John R. Goodlad, Tom C. Freeman, and Christopher D. Gregory

SUPPLEMENTAL FIGURES

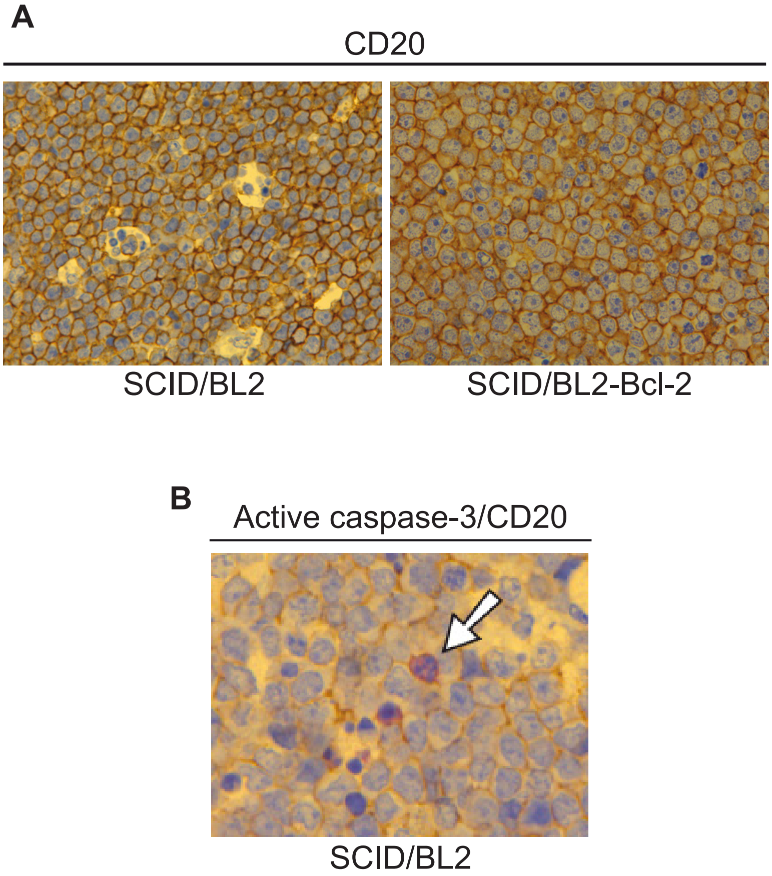

Figure S1, Related to Figure 1. Xenograft BL tumors principally contain human CD20^{+} cells and apoptotic tumor cells are CD20^{+}

(A) CD20 immunohistochemistry of BL2 (left) and BL2-Bcl-2 (right) xenograft tumors. Representative of at least 3 tumors of each type.

(B) BL2 xenograft tumor dual-stained for active caspase-3 (purple) and human CD20 (brown). Representative of at least 4 tumors.

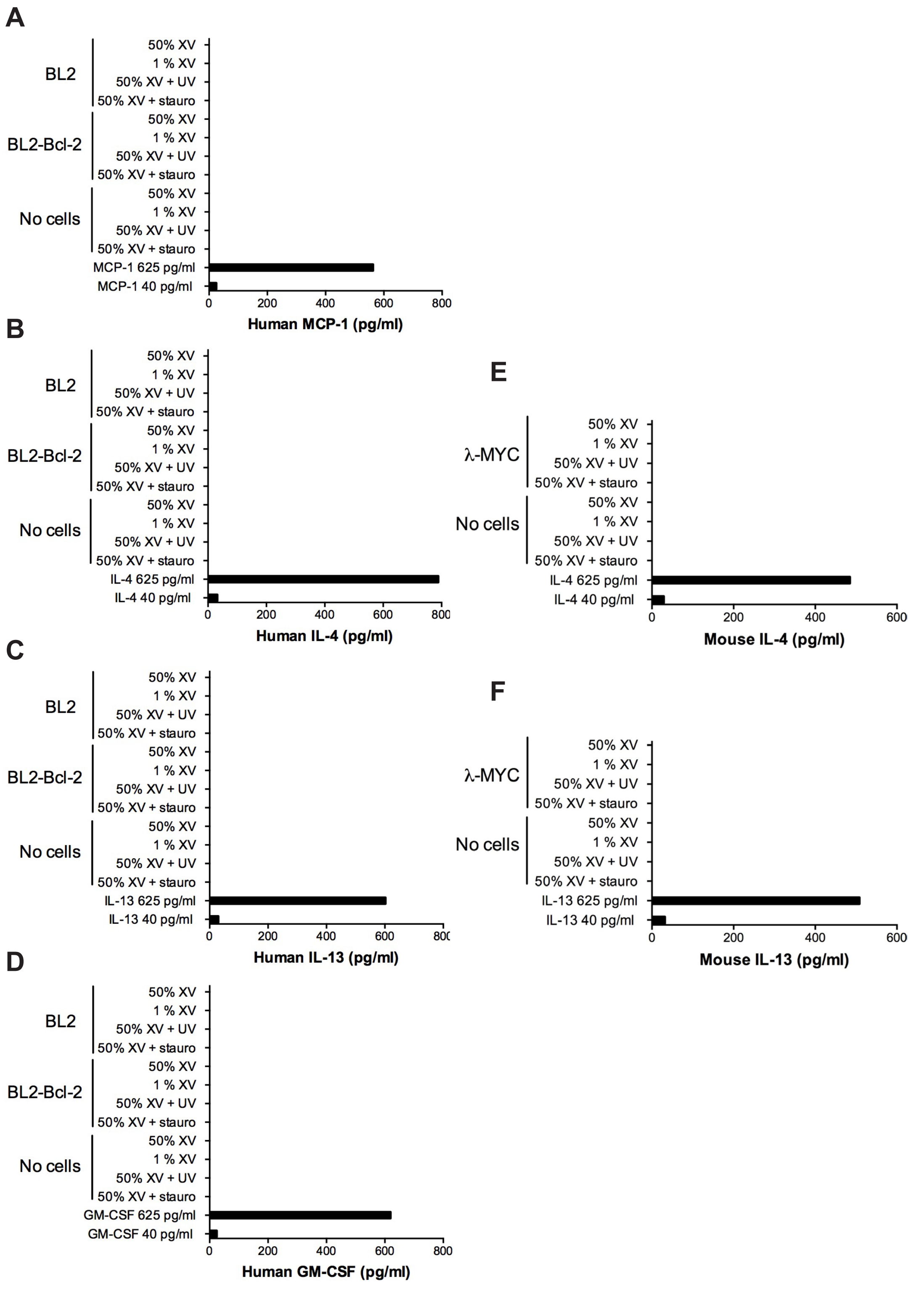

Figure S2, Related to Figure 2. Cytokine Bead Array (CBA) analysis of NHL supernatants.

BL2, BL2-Bcl-2 and λ-MYC cells cultured at 1 x 10^6/ml for 18 h in optimal (50% X-VIVO 20, Lonza) or sub-optimal (1% X-VIVO 20) conditions or 18 h following the induction of apoptosis by UV-irradiation (100 mJ/cm^2) or 1 µM staurosporine. For all treatments, human cytokines (**A**, MCP-1; **B**, IL-4; **C**, IL-13; **D**, GM-CSF) in BL2 or BL2-

Bcl-2 cell supernatant or mouse cytokines (**E**, IL-4; **F**, IL-13) in λ-MYC cell supernatant were undetectable. Cell culture medium spiked with a high and low concentration of the relevant cytokine (625 pg/ml and 40 pg/ml, respectively) was used as a positive control. Production of human IL-4, IL-13, GM-CSF, and MCP-1 (CCL2) was determined by a 4-plex CBA (BD), production of mouse IL-4 and IL-13 by a 2-plex CBA (BD) and production of mouse IL-6 and TNFα by a CBA mouse inflammation kit (BD). The lower limit of detection was 20 pg/ml.

These results suggest that inhibition of TAM accumulation in apoptosis-suppressed tumors seems unlikely to have been caused by Bcl-2/Bcl-x_L-induced alterations in cytokine/chemokine profiles occurring independently of the effects of Bcl-2/Bcl-x_L on apoptosis.

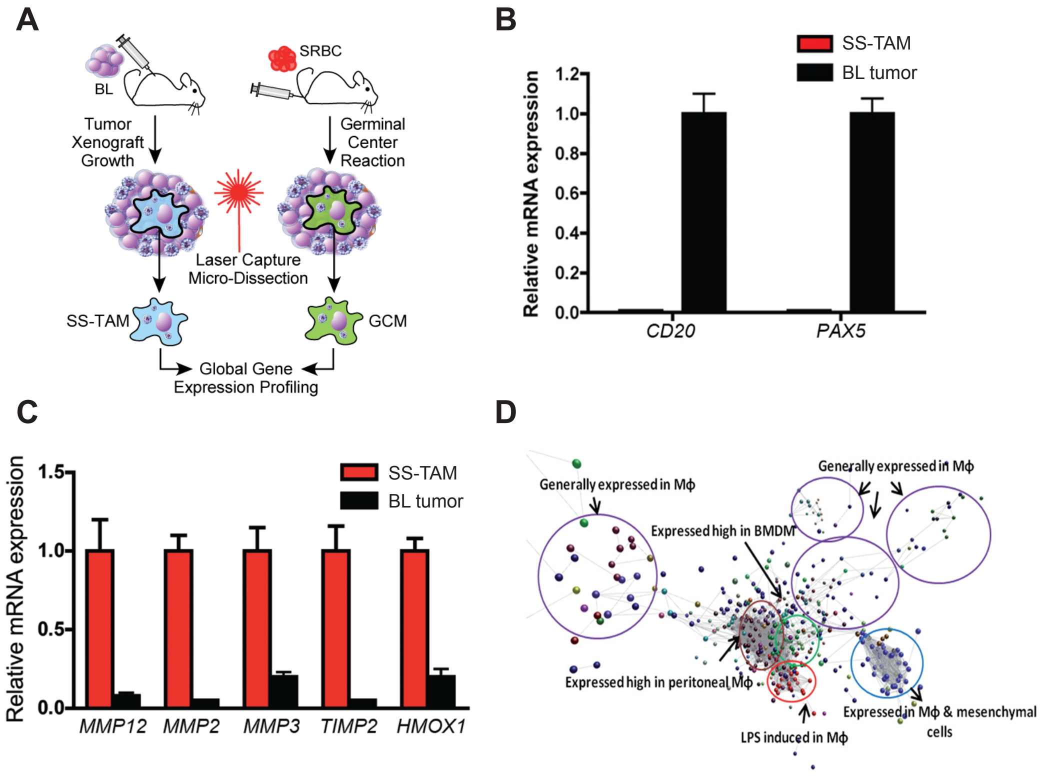

E

Annotation Cluster	Gene Count	Fold↑	P value
**Cluster 1 (groups 1-4: Extracellular matrix proteins)**		**7.55**	
-Group 1: Extracellular matrix	26		$7.3e^{-9}$
**Cluster 2 (groups 1-5)**		**5.35**	
-Group 1: Phagocytosis	8		$1.1e^{-5}$
**Cluster 3 (groups 1-10)**		**3.84**	
-Group 1: Chemotaxis	13		$3.7e^{-6}$
-Group 2: Inflammatory Response	7		$7.7e^{-5}$
**Cluster 4 (groups 1-18)**		**3.67**	
-Group 1: Wounding Response	32		$6.5e^{-13}$
-Group 2: Immune Response	33		$1.5e^{-7}$
-Group 3: Stress Response	41		$6.4e^{-7}$
-Group 4: Defense Response	33		$4.6e^{-5}$

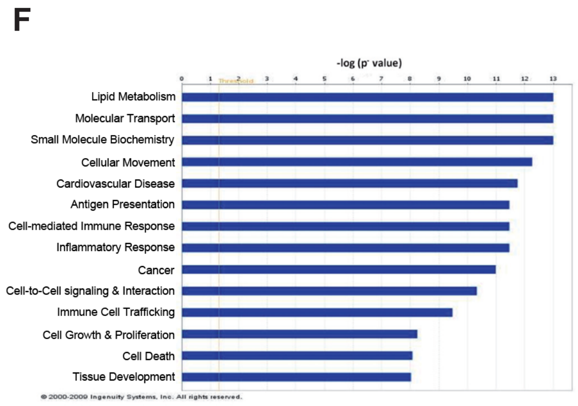

Figure S3, Related to Figure 4. Laser-capture microdissection of SS-TAMs, approach, validation and analysis.

(A) Schematic overview of "*in situ transcriptomics*" of SS-TAMs and germinal center macrophages (GCMs).

(B) SS-TAM mRNA does not contain transcripts for B-cell-specific markers (*CD20* and *PAX5*). Bars indicate relative mRNA expression (as assessed by qPCR) in laser micro-dissected TAMs (red) in comparison with whole BL tumor xenograft tissue (black). Data normalized to the housekeeping gene *TUBA1B* and presented as expression relative to whole BL tumor sample. Means +95% confidence intervals for n=2.

(C) Enrichment of selected transcripts in SS-TAMs with functional potential in matrix remodeling and angiogenesis. Bars indicate relative mRNA expression (as assessed by qPCR) in whole BL tumor xenograft tissue (black) in comparison with laser micro-dissected SS-TAMs (red). Data normalized to the housekeeping gene *TUBA1B* and presented as expression relative to TAM sample. Means +95% confidence intervals for n=2.

These results demonstrate that the gene profiling of laser-captured SS-TAMs was free from substantial RNA contamination caused by engulfed or co-captured tumor cells. Further selectivity of the profiling was likely due to the species differences between SS-TAMs (mouse) and tumor cells (human).

(D) Network analysis of SS-TAM transcriptome using the BioLayout *Express*^{3D} tool. The BioLayout *Express*^{3D} tool was used for network analysis of the TAM transcriptome. Briefly, the software generates and analyses network graphs from large microarray gene expression datasets by clustering together sets of genes with similar expression patterns as calculated by a Pearson correlation coefficient using the MCL cluster algorithm [S1]. The mouse transcriptomics atlas (GNFv3 cell atlas) used for validation of TAM transcriptome was generated in BioLayout *Express*^{3D} as described [S2]. Significantly up-regulated genes were compared with the GNFv3 Mouse Cell Atlas network [S2]. The network graph comprises nodes that represent transcripts, and edges that represent connections based on Pearson correlation coefficients of 0.8 or greater. Node colors represent assigned clusters. The vast majority of differentially expressed TAM transcripts were identified as macrophage-specific as shown by the annotated clusters.

(E) Functional clustering of enriched SS-TAM transcripts using The Database for Annotation, Visualization and Integrated Discovery (DAVID, http://david.abcc.ncifcrf.gov) functional annotation clustering online tool. Enrichment scores for the four most highly enriched SS-TAM clusters are shown. Each of the clusters comprises similar GO terms presented as functional groups. Gene counts and P-values for each of the groups are shown. Data from 3 animals, significance analyzed using the Benjamini and Hochberg procedure. Matrix remodeling, phagocytosis and response to wounding are highlighted in SS-TAM following functional analysis by DAVID.

(F) Biological processes and functions enriched in SS-TAMs. All significantly up-regulated SS-TAM genes were analyzed for biological processes and molecular/cellular functions using Ingenuity Systems Pathway Analysis (IPA). Lipid metabolism, inflammatory response, proliferation and development are among the processes highlighted by IPA analysis.

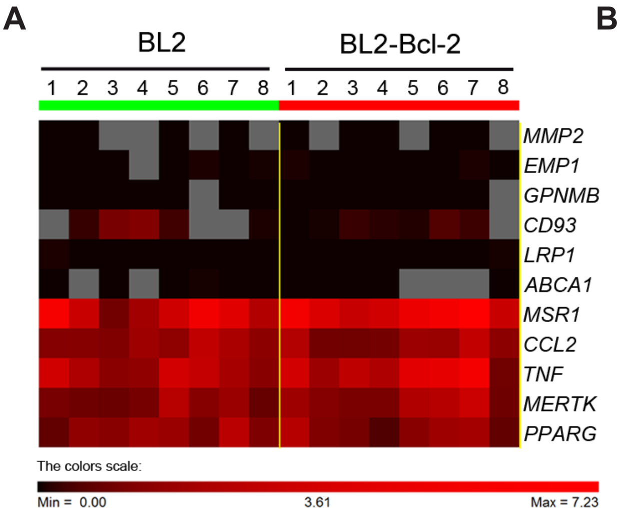

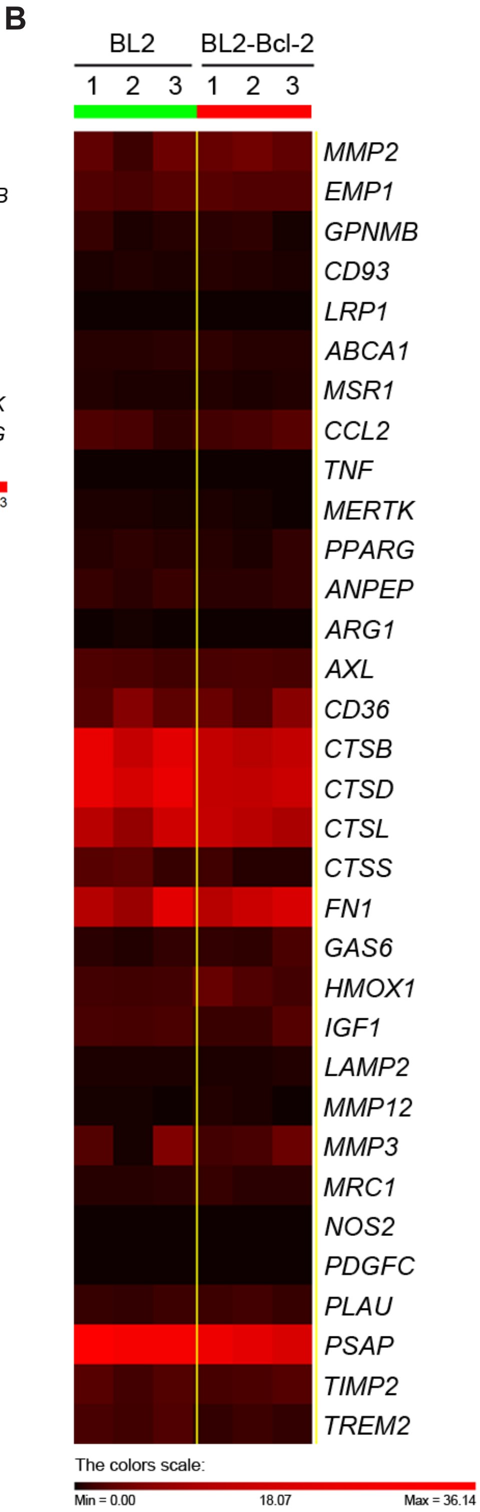

Figure S4, Related to Figure 4. TAMs in apoptosis-suppressed xenograft tumors show similar gene expression profile to their parental counterparts

(A) Heat map showing Fluidigm gene expression analysis of genes in BL2 compared to BL2-Bcl-2 xenograft formalin-fixed, paraffin-embedded tumor tissue (n=8 for each tumor type). Data were normalized to *EMR1* gene expression.

(B) Heat map comparing murine mRNA expression (analyzed by qPCR and normalized to the myeloid gene *CSF1R*) of selected genes in frozen tumor biopsies of BL2 and BL2-Bcl-2 xenografts (n=3 for each tumor type).

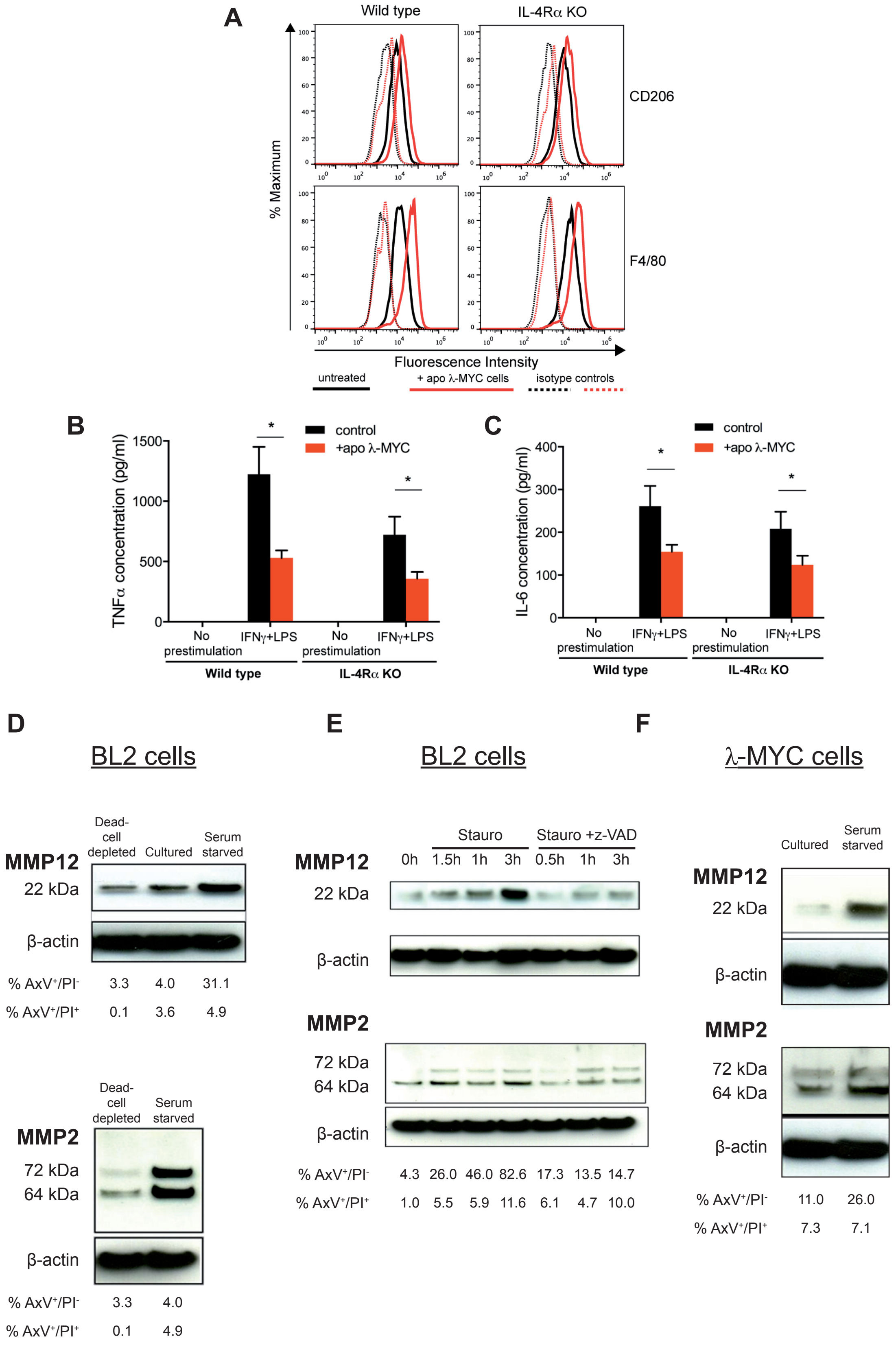

Figure S5, Related to Figure 5. Apoptotic lymphoma cells activate metalloproteinases and promote pro-tumor gene expression in macrophages *in vitro*.

(A-C) Wild-type (WT) versus IL-4Rα knockout (KO) BMDM responses to apoptotic λ-MYC cell co-culture. BMDMs from WT or IL-4Rα KO C57BL/6 mice were co-cultured for 24 h with or without apoptotic λ-MYC cells. Apoptosis of λ-MYC cells was triggered by 100 mJ/cm^2 UVB-irradiation. **(A)** BMDMs were assessed for cell surface expression of CD206 and F4/80 by flow cytometry. TNFα **(B)** and IL-6 **(C)** release following apoptotic λ-MYC cell co-culture. Some BMDMs were classically activated (IFN-γ+LPS) prior to co-culture with apoptotic λ-MYC cells. Means + SEM for 5 independent experiments. Wilcoxon matched pairs test **(B)** * $P<0.05$ WT IFN-γ+LPS control and WT IFN-γ+LPS + apo λ-MYC, * $P<0.05$ IL-4Rα KO IFN-γ+LPS control and IL-4Rα KO IFN-γ+LPS + apo λ-MYC; **(C)** * $P<0.05$ WT IFN-γ+LPS control and WT IFN-γ +LPS + apo λ-MYC, * $P<0.05$ IL-4Rα KO IFN-γ+LPS control and IL-4Rα KO IFN-γ+LPS + apo λ-MYC. This combination of flow cytometric and qPCR analyses of BMDMs indicated that co-culture with apoptotic λ-MYC cells could promote the expression of a variety of SS-TAM markers in BMDMs including CD206, F4/80, *CTSL*, *PDGFC* and *HMOX1* (see also Figure 5I). Note that the changes were small in BMDMs which we believe reflects the tendency of the BMDM activation status to be constitutively polarized towards that of SS-TAMs, most likely as a result of apoptosis occurring during the course of BMDM differentiation in culture in the presence of CSF-1 (our unpublished observations).

(D) Immunoblots showing reduced expression of MMP12 (22kDa) and MMP2 (64; 72 kDa) polypeptides following apoptotic-cell depletion, and enhanced expression following apoptosis induction (1 h serum-starvation), in BL2 cells (representative of n=3 independent experiments).

(E) Immunoblots showing enhancement of MMP12 and MMP2 expression/processing in staurosporine-treated (apoptotic) but not z-VAD-treated (viable) BL2 cells (representative of n=3 independent experiments).

(F) Enhanced expression of MMP12 and MMP2 polypeptides following apoptosis induction (1 h serum-starvation), in λ-MYC cells (representative of n=3 independent experiments). Percentages of apoptotic (AxV^+/PI^-) and dead (AxV^+/PI^+) cells for each condition are shown.

These results demonstrate that apoptotic lymphoma cells can directly express and process these MMPs.

SUPPLEMENTAL METHODS: DETAILED EXPERIMENTAL PROCEDURES

Cells and Tumors

BL lines and THP-1 cells were cultured in RPMI 1640, THP-1 cells with phorbol 12-myristate 13-acetate (200 nM, Sigma) for 72 hr. Bcl-2 and Bcl-x_L transfectants were derived as described [S3]. RAW264.7 cells were cultured in high glucose DMEM. The mouse λ-MYC line was derived from a spontaneous tumor of a C57BL/6 λ-MYC mouse [S4] and was maintained in high glucose DMEM:IMDM (1:1) growth medium with 25 µM β-mercaptoethanol. Bone marrow-derived macrophages (BMDMs) from WT or IL-4Rα KO C57BL/6 mice were harvested from femurs and cultured in RPMI 1640 supplemented with 100 ng/ml rhM-CSF (R&D Systems) on bacteriological-grade Petri dishes. In some *in vitro* co-cultures, BMDMs were classically activated using 10 U/ml murine IFN-γ (R&D Systems) and 0.5 ng/ml LPS (Sigma) for 4 h prior to co-culture. All media were supplemented with 10% FCS, 2 mM L-glutamine, 100 U/ml penicillin and 100 µg/ml streptomycin. Human monocytes were prepared from the blood of healthy donors (Lothian Research Ethics Committee approvals no. 08/S1103/38) as described [S5]. Human monocyte-derived macrophages (HMDMs) were matured from isolated blood monocytes by 7-day culture in IMDM supplemented with 10% heat-inactivated autologous serum, 2 mM L-glutamine, 100 U/ml penicillin and 100 µg/ml streptomycin. Xenograft tumors were generated by s.c. injection of 10×10^6 BL2, BL2-Bcl-2, or BL2-Bcl-x_L or i.p. injection of 10×10^6 Mutu I or Mutu-Bcl-2 cells into 6-12 week-old male and female BALB/c SCID mice. For macrophage-depletion experiments, 200 µl clodronate- or PBS (control)- containing liposomes [S6] were injected daily i.v. following detection of s.c. tumor for 4 days, with tumors being harvested 24 h after the final liposome dose. λ-MYC tumors were generated by s.c. injection of 5×10^6 viable λ-MYC cells – used either after depletion of apoptotic cells or after mock-depletion – into 6-12 week-old male WT or IL-4Rα KO C57BL/6 mice. Apoptotic cells were depleted by magnetic separation as described [S7]. Mock-depleted cells were subjected to the same procedure in the absence of apoptotic-cell binding magnetic microparticles. Depleted cells were 94% viable and mock-depleted cells 62% viable by trypan blue exclusion. Syngeneic mouse melanoma development was studied by s.c. injection of a sub-tumorigenic dose of viable B16 cells (6×10^4) into 5-7 week old C57BL/6 mice. B16 cells were co-injected with 10^4-10^7 irradiated (75 Gy) B16 cells or with 5×10^5 peritoneal macrophages from naïve C57BL/6 mice. Macrophages were cultured for 4 h before co-injection. Alternatively C57BL/6 mice were injected s.c. with 1×10^5 λ-MYC cells alone or co-injected along with 1×10^7 UV-irradiated (200 mJ/cm^2) λ-MYC cells. 1×10^7 UV-irradiated cells alone were injected s.c. in a control group of mice. Growth of tumors was monitored using calipers. In all experiments, mice were humanely sacrificed when tumors reached dimensions equivalent to 1.44 cm^2. All animal procedures and husbandry were performed under a license from the UK Home Office according to regulations described in the Animal (Scientific Procedures) Act 1986. Cell lines and transfectants were negative for mycoplasma species by VenorGeM Detection Kit (Cambio (Minerva BioLabs)). Formalin-fixed, paraffin-embedded archival human BL tumor tissue was obtained and used with ethical committee approval (North West 5 Research Ethics Committee, Haydock Park (Ref 09/H1010/75)).

Histology and immunohistochemistry

Tissue sections were stained with standard H&E or were used in IHC as described [S8]. Formalin-fixed, paraffin-embedded tissue was probed with antibodies to human Ki67 (Leica (Novocastra Laboratories) NCL-Ki67p), human CD20 (clone L26, Dako), human

CD68 (clone PG-M1, Dako), active caspase 3 (Millipore (Calbiochem) or Cell Signaling Technology), HIF-1α (clone ESEE122, Novus Biologicals) or mouse Ki67 (Bethyl Laboratories) following heat treatment in citric acid-based antigen retrieval solution. Mouse-on-mouse blocking kit (Vector Laboratories) was used with anti-CD20 and anti-HIF-1α antibodies. Apoptosis was also detected with TUNEL using ApopTag peroxidase *in situ* apoptosis detection kit (Millipore). Frozen mouse tissue was stained with antibodies to CD31 (clone 390, BioLegend), mouse CD68 (clone FA-11, Serotec), CD169 (clone 3D6.112, Serotec), CD206 (clone MR5D3, Serotec) and F4/80 (clone Cl:A3-1, Serotec). Methacarn-fixed, paraffin embedded tissue was probed with anti-F4/80 (clone BM8, Life Technologies) without antigen retrieval. Methacarn-fixed tissue was dual stained with anti-F4/80 (clone BM8) and anti-mouse Ki67 with Borg decloaker antigen retrieval (A. Menarini Diagnostics (MenaPath)). All IHC staining was detected using biotinylated secondary antibodies (Vector) and DAB (Vector) except dual stained tissue, which used Alexa Fluor 546 (Life Technologies) for Ki67 and Tyramide TSA-Plus Fluorescein (Perkin-Elmer) for F4/80 detection or VIP peroxidase (Vector) for active caspase 3 and DAB (Vector) for mouse CD206 and human CD20. Dual staining was visualized with a Zeiss LSM 510 confocal microscope. Systematic random sampling was used for morphometric analyses.

Analysis of apoptosis, proliferation and cell cycle *in vitro*
Apoptosis was induced by exposure of cells to staurosporine (1 µM, Millipore), by culture at high density (100 x 10^6/ml) for 1 h in serum-free medium, or by exposure to 100 or 200 mJ/cm^2 UVB irradiation. Apoptosis was blocked using z-VAD-fmk (100 µM, R and D Systems). Apoptosis was assessed using annexin V (AxV, Life Technologies) binding in combination with propidium iodide (PI, Life Technologies) staining [S9]. Proliferation of BL cells cultured under normoxic (21% CO_2) or hypoxic (0.5% CO_2) conditions or co-cultured with RAW264.7 (normoxia) for up to 72 h was assessed by fixing cells in 10% formaldehyde in PBS with 5% BSA followed by analysis on an EPICS XL flow cytometer (Beckman Coulter) along with a known number of Flow-Count fluorospheres (Beckman Coulter) [S10, S11]. Flow cytometric cell cycle analysis was performed on fixed (2% formaldehyde) and permeabilized (0.2% saponin) cells stained with PI (50 µg/ml) [S11]. Sub-diploid cells were excluded by electronic gating. All *in vitro* analyses were performed at least 3 independent times.

Immunoblotting
Cell lysates were prepared and used in immunoblotting as described [8]. Membranes were probed with antibodies to MMP2 (Santa Cruz Biotechnology), MMP12 (Abcam) and β-actin (clone AC-15, Sigma).

Macrophage *in situ* gene expression profiling
Laser-capture microdissection was performed on frozen 10 µm SCID/BL2 tissue sections stained with rapid one-step immunolabeling using anti-CD68 AlexaFluor-488 antibody (Life Technologies) with Protector RNase Inhibitor (4 U/ml, Roche) or splenic germinal centers from *CSF1R EGFP* transgenic BALB/c mice stimulated with two doses of 5 x 10^8 sheep red blood cells (SRBC). A PALM MicroBeam system (Palm Microlaser Technologies) was used to cut out tumor-associated CD68$^+$ or germinal center macrophages. Captured cells (1000 cells) were lysed and RNA extracted using the RNeasy Plus Micro Kit (Qiagen). RNA yield and quality was assessed using the RNA6000 Pico Assay on an Agilent Bioanalyzer (Agilent Technologies) and total RNA was amplified and cDNA synthesized for whole gene expression analysis using

WT-Ovation Pico RNA Amplification Systems (NuGEN Technologies). Amplified cDNA was processed for sense-transcript cDNA generation using WT Ovation Exon Module (NuGEN Technologies) and fragmentation/labeling using FL-Ovation cDNA Biotin Module V2 (NuGEN Technologies) for gene microarray hybridization on Affymetrix Mouse Gene 1.0 GeneChip arrays as per Affymetrix instructions.

Fluidigm gene expression analysis was performed on RNA purified from 16 x 10 μm sections from formalin-fixed paraffin-embedded xenograft tumor using RecoverAll™ Total Nucleic Acid Isolation Kit (Life Technologies), as per the manufacturer's protocol. The amount and quality of RNA extracted was assessed using a NanoDrop Bioanalyzer (ND1000, Celbio). Complementary DNA (cDNA) was generated using SuperScript III First-Strand Synthesis Supermix (Life Technologies). Preamplification reactions and real-time PCRs were performed using Fluidigm dynamic array gene expression technology (Fluidigm) following the protocol outlined in the Fluidigm advanced development protocol PN 100-1208 B1 using EvaGreen DNA binding dye and specific primers for murine macrophage genes. Following melt curve analysis the data were normalized using either *CD68* or *EMR1* (F4/80), to generate a ΔC_t value for each gene. Duplicate samples generally varied by no more than 5%. Expression values were then calculated as relative abundance with the formula $2^{-\Delta Ct}$. Heat maps were generated using PermutMatrix software.

Real time qPCR

Real time PCR cDNA was generated from total RNA from macrophages or snap-frozen tumor biopsies using SuperScript III First-Strand Synthesis SuperMix (Life Technologies). Real time qPCR was performed in an ABI 7500 FAST qPCR system using SYBR Green I Dye chemistry (Life Technologies) and gene-specific primers. Relative mRNA expression for genes of interest was assessed in comparison to a calibrator sample, following normalization to α-tubulin (*TUBA1B*), *CSF1R*, *EMR1* or *CD68* using the ΔΔCT method [S12].

Flow cytometry

BMDM were assessed for cell surface expression of CD206 (anti-CD206-Alexa Fluor 488-conjugate, clone MR5D3, AbD Serotec) and F4/80 (anti-F4/80-Alexa Fluor 488-conjugate, clone BM8, Life Technologies) as described previously [S10].

***In vitro* co-culture**

Macrophage cell lines or primary macrophages (HMDMs or BMDMs) were seeded in tissue culture plates. BL2 and λ-MYC cell lines induced to undergo apoptosis were added to macrophages at a 1:10 macrophage to BL2/λ-MYC ratio. Following up to 24 h co-culture, supernatants were collected and cleared by sequential 300 x g and 1220 x g centrifugation steps for cytokine analysis. Adherent macrophages were washed repeatedly with PBS to completely remove BL2 or λ-MYC cells, before RNA was extracted using a RNeasy Mini Kit (Qiagen), protein was extracted for immunoblotting, or cells were harvested for analysis by flow cytometry.

SUPPLEMENTAL REFERENCES

S1. Freeman, T.C., Goldovsky, L., Brosch, M., van Dongen, S., Maziere, P., Grocock, R.J., Freilich, S., Thornton, J., and Enright, A.J. (2007). Construction, visualisation, and clustering of transcription networks from microarray expression data. PLoS Comput Biol *3*, 2032-2042.

S2. Hume, D.A., Summers, K.M., Raza, S., Baillie, J.K., and Freeman, T.C. (2010). Functional clustering and lineage markers: insights into cellular differentiation and gene function from large-scale microarray studies of purified primary cell populations. Genomics *95*, 328-338.

S3. Milner, A.E., Grand, R.J.A., Vaughan, A.T.M., Armitage, R.J., and Gregory, C.D. (1997). Differential effects of BCL-2 on survival and proliferation of human B-lymphoma cells following gamma-irradiation. Oncogene *15*, 1815-1822.

S4. Kovalchuk, A.L., Qi, C.F., Torrey, T.A., Taddesse-Heath, L., Feigenbaum, L., Park, S.S., Gerbitz, A., Klobeck, G., Hoertnagel, K., Polack, A., et al. (2000). Burkitt lymphoma in the mouse. Journal of Experimental Medicine *192*, 1183-1190.

S5. Truman, L.A., Ogden, C.A., Howie, S.E., and Gregory, C.D. (2004). Macrophage chemotaxis to apoptotic Burkitt's lymphoma cells in vitro: role of CD14 and CD36. Immunobiology *209*, 21-30.

S6. Van Rooijen, N., and Sanders, A. (1994). Liposome mediated depletion of macrophages: mechanism of action, preparation of liposomes and applications. J Immunol Methods *174*, 83-93.

S7. Gregory, C.D., Pound, J.D., Devitt, A., Wilson-Jones, M., Ray, P., and Murray, R.J. (2009). Inhibitory effects of persistent apoptotic cells on monoclonal antibody production in vitro: simple removal of non-viable cells improves antibody productivity by hybridoma cells in culture. MAbs *1*, 370-376.

S8. Truman, L.A., Ford, C.A., Pasikowska, M., Pound, J.D., Wilkinson, S.J., Dumitriu, I.E., Melville, L., Melrose, L.A., Ogden, C.A., Nibbs, R., et al. (2008). CX3CL1/fractalkine is released from apoptotic lymphocytes to stimulate macrophage chemotaxis. Blood *112*, 5026-5036.

S9. Devitt, A., Parker, K.G., Ogden, C.A., Oldreive, C., Clay, M.F., Melville, L.A., Bellamy, C.O., Lacy-Hulbert, A., Gangloff, S.C., Goyert, S.M., et al. (2004). Persistence of apoptotic cells without autoimmune disease or inflammation in CD14-/- mice. J Cell Biol *167*, 1161-1170.

S10. Ogden, C.A., Pound, J.D., Batth, B.K., Owens, S., Johannessen, I., Wood, K., and Gregory, C.D. (2005). Enhanced Apoptotic Cell Clearance Capacity and B Cell Survival Factor Production by IL-10-Activated Macrophages: Implications for Burkitt's Lymphoma. J Immunol *174*, 3015-3023.

S11. Milner, A.E., Levens, J.M., and Gregory, C.D. (1998). Flow cytometric methods of analyzing apoptotic cells. Methods Mol Biol *80*, 347-354.

S12. Livak, K.J., and Schmittgen, T.D. (2001). Analysis of relative gene expression data using real-time quantitative PCR and the 2(-Delta Delta C(T)) Method. Methods *25*, 402-408.
